# New and old lessons from a devastating case of neonatal *E coli* meningitis

**DOI:** 10.1186/s12887-024-04787-y

**Published:** 2024-05-16

**Authors:** Tawny Saleh, Edwin Kamau, Jennifer A. Rathe

**Affiliations:** 1 Department of Pediatrics, Division of Infectious Diseases, David Geffen School of Medicine at UCLA, Los Angeles, CA USA; 2 Department of Pathology and Laboratory Medicine, David Geffen School of Medicine at UCLA, Los Angeles, CA USA; 3 Present address: Department of Pathology and Area Laboratory Services, Tripler Army Medical Center, Honolulu, HI USA

**Keywords:** Neonatal *E coli* meningitis, Acridine orange stain, Neonatal sepsis, CSF sterility

## Abstract

**Background:**

Neonatal *Escherichia coli* (*E coli*) meningitis results in significant morbidity and mortality. We present a case of a premature infant with extensive central nervous system (CNS) injury from recurrent *E coli* infection and the non-traditional methods necessary to identify and clear the infection.

**Case Presentation:**

The infant was transferred to our institution’s pediatric intensive care unit (PICU) after recurrence of *E coli* CNS infection requiring neurosurgical intervention. He had been treated for early onset sepsis (EOS) with ampicillin and gentamicin for 10 days followed by rapid development of ampicillin-resistant *E coli* septic shock and meningitis after discontinuation of antibiotics. Sterility of the CNS was not confirmed at the end of 21 days of cefepime therapy and was subsequently followed by recurrent ampicillin-resistant *E coli* septic shock and CNS infection. Despite 6 weeks of appropriate therapy with sterility of CSF by traditional methods, he suffered from intractable seizures with worsening hydrocephalus. Transferred to our institution, he underwent endoscopic 3rd ventriculostomy with cyst fenestration revealing purulent fluid and significant pleocytosis. An additional 3 weeks of systemic and intraventricular antibiotics with cefepime and tobramycin were given but a significant CNS neutrophil-predominant pleocytosis persisted (average of $\sim$ 21,000 cells/mm^3^). Repeated gram stains, cultures, polymerase chain reaction (PCR) testing, and metagenomic next generation sequencing (NGS) testing of CSF were negative for pathogens but acridine orange stain (AO) revealed numerous intact rod-shaped bacteria. After the addition of ciprofloxacin, sterility and resolution of CSF pleocytosis was finally achieved.

**Conclusion:**

Neonatal *E coli* meningitis is a well-known entity but unlike other bacterial infections, it has not proven amenable to shorter, more narrow-spectrum antibiotic courses or limiting invasive procedures such as lumbar punctures. Further, microbiologic techniques to determine CSF sterility suffer from poorly understood limitations leading to premature discontinuation of antibiotics risking further neurologic damage in vulnerable hosts.

## Introduction

Neonatal *E coli* sepsis results in high morbidity and mortality due to complex host and environmental factors, but early identification and appropriate treatment limits progression to meningitis and/or significant parenchymal damage (encephalitis). Recurrence or relapse of CNS infection is a well-recognized entity of neonatal *E coli* meningitis with increased risk associated with the K1 virulence factor and signs of complicated infection (abnormalities on brain imaging: ventriculitis, abscess or clinical signs of CNS infection: seizures or altered mental status). Therefore, repeat LP to document sterility prior to the end of antibiotics has been a longstanding recommendation [1, 2].

Neonatal EOS presents within the first 72 h of life [3]. Late onset sepsis (LOS) presents in the first 4 to 28 days of life. EOS pathogens colonize the infant as they pass through the vaginal canal. Risk of EOS increases with prolonged rupture of membranes and maternal fever or infection. Conversely, LOS pathogens are obtained from the neonates’ environment with significantly increased risk of severe infection for very low birthweight premature infants. Group B Streptococcal (GBS) and *E coli* are the most common pathogens responsible for EOS, with *E coli* rates rising, while a variety of gram positive and negative species are responsible for LOS without a particular pathogen predominance [3, 4]. Empiric therapy for EOS is generally ampicillin and gentamicin [3]. If meningitis is a concern, cefotaxime or cefepime are recommended with ampicillin; gentamicin has limited CNS penetration due to its large, hydrophilic nature. Consequently, aminoglycosides can be used as an adjunct but are not reliable as the primary treatment for meningitis [3, 5]. Recommendations for empiric antibiotic therapy for LOS now include a broad-spectrum 3rd or 4th generation cephalosporin +/- vancomycin due to the wide range of causative bacterial organisms and concerns for highly pathogenic gram-negative organisms [3, 6]. However, recommendations for empiric therapy for EOS continue to be ampicillin and gentamicin.

## Case presentation

A 3-month-old male born prematurely at 27 weeks gestation was transferred to our institution with recurrent *E coli* CNS infection. He was born via vaginal delivery after premature and prolonged rupture of membranes, with unremarkable prenatal labs. Within 48 h, the neonate became unstable with concern for EOS but blood cultures were negative; he was treated for culture negative sepsis with 10 days of ampicillin and gentamicin.

**Fig. 1:**
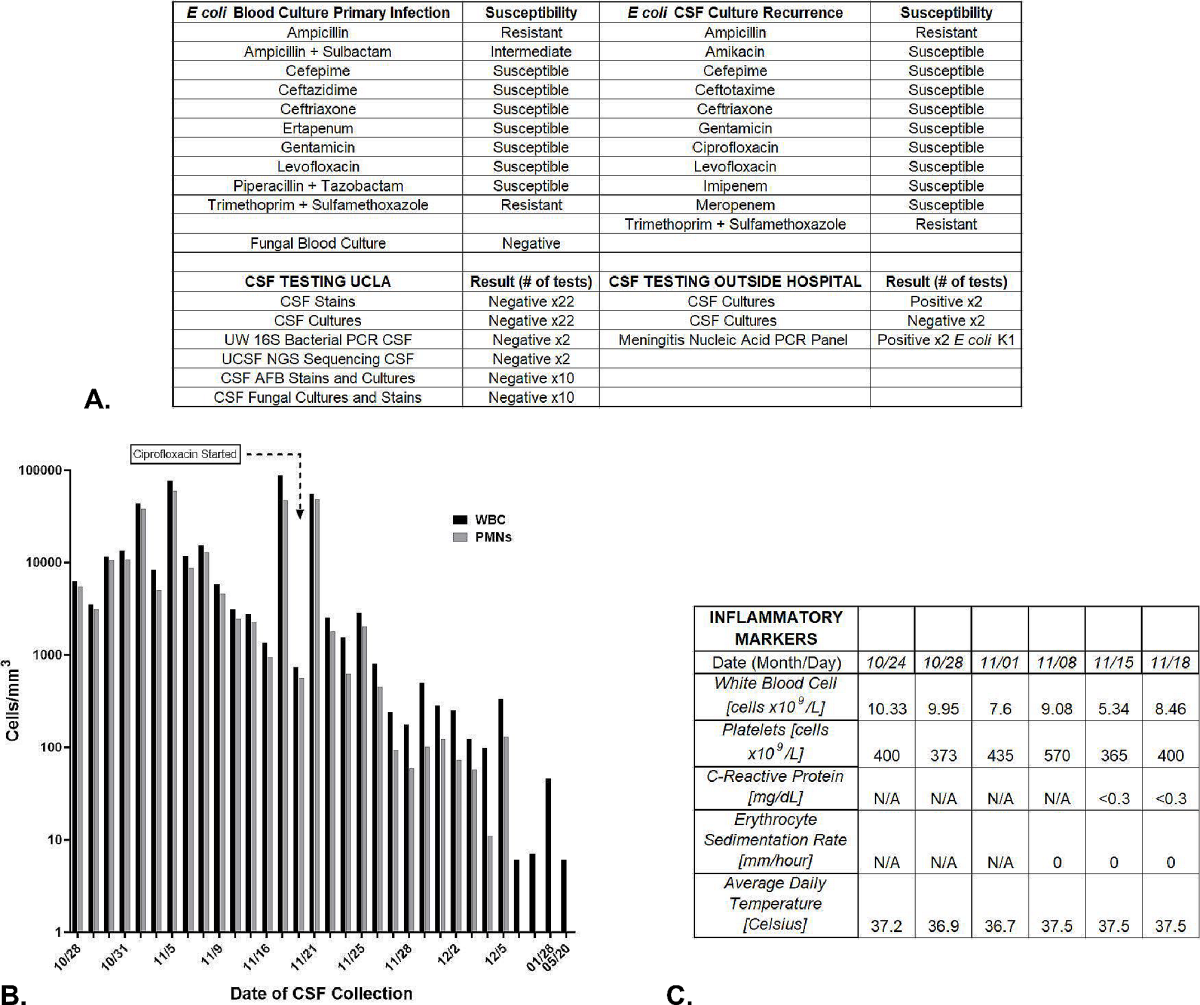
Cerebral Spinal Fluid (CSF), Serum, and Clinical Markers of Inflammation. **A**: Culture and susceptibility testing results from blood and CSF samples from the outside hospitals from the first and recurrent episode of *E coli* infection. Culture and testing results from UCLA after transfer. **B**: This graph demonstrates the pleocytosis measured in the CSF over the course of antibiotic treatment during the patient’s admission. Black bars demonstrate the levels of WBCs detected on sampling and the grey bars represent the proportion of those WBCs consisting of neutrophils. The start of ciprofloxacin is shown with a text box and arrow at the top of the graph. Neutrophil predominant CSF pleocytosis resolved after the introduction of ciprofloxacin. **C**: Serum and core temperature measurements of the patient at the time frame concerning for continued infection despite negative CSF gram stain, cultures, bacterial 16 S PCR testing, and unbiased pathogen NGS testing. All testing was negative for signs of systemic inflammation

Within a week after completion of antibiotics, he went into septic shock with ampicillin-resistant *E coli* bacteremia (Fig. 1A) complicated by intractable seizures, grade 3–4 intraventricular hemorrhage (IVH), and respiratory failure requiring intubation. Lumbar puncture (LP) was not performed secondary to patient instability. Cefepime was administered for 21 days for presumed *E coli* meningitis with some clinical improvement but seizures continued. Brain magnetic resonance imaging (MRI) and serial head ultrasounds were significant for global encephalomalacia and worsening hemorrhage with ventriculomegaly. No LP was performed at the end of therapy.

Two weeks after completion of antibiotics, the patient again went into septic shock with ampicillin-resistant *E coli* bacteremia, meningitis, and ventriculitis. He was treated with cefepime and gentamicin. He was diagnosed via a positive blood culture and positive CSF cultures (same sensitivities, Fig. 1A). Two subsequent CSF samples were negative by culture and stain, but were positive via a meningitis nucleic acid PCR panel for *E coli* after 5 and 6 weeks of therapy. Inflammatory markers and clinical signs of sepsis normalized by the 6th week of therapy with the exception of seizures. An MRI revealed multiple heterogenous cystic structures concerning for hydrocephalus as well as loculated areas consistent with infection and/or hemorrhage. After 8 weeks of antibiotics, he was experiencing increasing hydrocephalus and seizures; he was transferred to our institution for neurosurgical intervention.

Neurosurgery performed an endoscopic 3rd ventriculostomy with cyst fenestration and reservoir placement. The surgeons encountered diffuse, thick, purulent material in the accessible intra-parenchymal and -ventricular cysts. CSF studies demonstrated significant pleocytosis with a high percentage of neutrophils, but gram stain and cultures were negative. Cefepime was continued with the addition of systemic and intraventricular (IT) tobramycin. Despite an aggressive antibiotic regimen for 3 weeks (+ 8 weeks prior to transfer), serial CSF sampling continued to demonstrate significant neutrophil-predominant pleocytosis (Fig. 1B). However, gram stains and cultures of the CSF remained negative. Further, metagenomic NGS tests performed at University of California San Francisco and 16 S bacterial long-range PCR performed at University of Washington did not detect any pathogenic genomic material. Systemic inflammatory markers were normal and clinical symptoms of infection were absent (Fig. 1C).

**Fig. 2:**
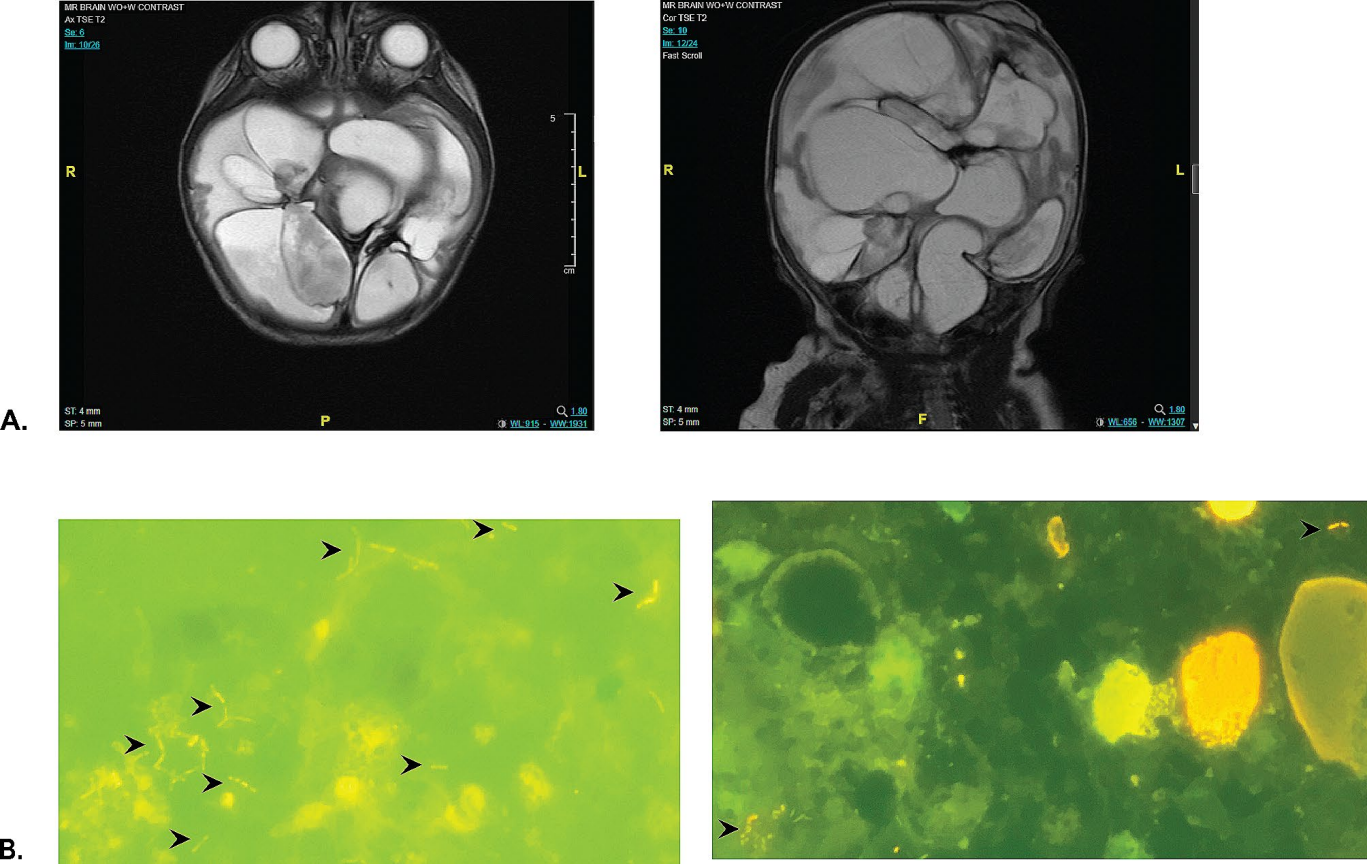
MRI Brain Imaging and Acridine Orange (AO) Stains of CSF Samples Prior to Ciprofloxacin. **A**: MRI brain imaging of the patient axial and coronal views. Multiple discontinuous fluid filled cysts are noted throughout the brain with minimal remaining brain parenchyma. **B**: Sections of representative microscopic slides of patient’s CSF AO stains of two separate CSF collections obtained 1–2 days apart. Black arrows locate the rod-shaped intact organisms. The connected rods are consistent with images of actively dividing organisms such as *E. coli*

It was unclear if the pleocytosis was due to inflammation from significant brain damage or continued *E coli* infection. Brain MRI demonstrated the presence of multiple, non-communicating cysts (Fig. 2A). We hypothesized that cultures were negative not because the CNS was sterile but rather that there were sequestered areas of infection in the non-communicating brain cysts and the instillation of tobramycin may be affecting culture results. The clinical microbiology lab recommended an alternative stain with AO to help visualize any organisms that might be present. Assessment of several CSF samples with AO found multiple, rod-shaped, intact bacterial organisms (Fig. 2B). None of the associated gram stains, cultures, PCR, nor NGS testing of these samples detected microorganisms. With concern for possible development of antibiotic resistance, a new secondary infection, and improved CNS penetration, we discontinued tobramycin and started ciprofloxacin with continuation of cefepime. Previous cultures demonstrated *E coli* sensitivity to ciprofloxacin (Fig. 1A). Four weeks after the addition of ciprofloxacin, the CSF pleocytosis resolved and AO stains were negative. A ventricular peritoneal shunt was placed, and he has remained infection free for > 2 years.

Given his recurrent infection and significant CNS damage, he was evaluated for a primary immune deficiency by Allergy and Immunology; the work up did not identify abnormalities on lymphocyte panel, quantitative immunoglobulins, CH50 testing, TLR function, etc. He is now > 2 years old. He has severe global developmental delays: he is blind, he speaks no words, he has mild hearing loss, he cannot support his head, roll over, crawl, nor use his limbs in purposeful movements, and lastly, he startles to sounds but does not respond to his name nor commands.

## Discussion and conclusions

Bacterial meningitis in neonates and infants is a rare occurrence; incidence is estimated at $\sim$ 1–2% for full term infants and 4–6% for preterm/very low birth-weight neonates [3, 4]. However, EOS and LOS are far more common clinical presentations, especially in preterm infants, with the risk of progression to meningitis. As appreciated in this case, meningitis in these populations have a high likelihood of resulting in some amount of neurodevelopmental injuries with a range of severity [7, 8]. Our patient experienced EOS and LOS in rapid succession suggesting that both instances resulted from infection with the same ampicillin-resistant *E coli.* In other words, his EOS was treated with gentamicin alone and may have predisposed to his LOS either because of partially treated *E coli* CNS infection or as the result of a new infection from translocation of colonizing *E coli* in his leaky, premature gastrointestinal tract. The high rates of *E coli* resistance increasing globally raises concerns about the safety of continued empiric use of ampicillin and gentamicin in EOS [9]. However, given the large number needed to treat secondary to the rare incidence of EOS progressing to bacterial meningitis, it is not clear that the subsequent trade off of increased antibiotic resistance is worth the small number of patients that would benefit from decreased morbidity and mortality. At this time, many argue the change is not warranted and that individual risk assessment and local antibiotic resistance rates are key in the decision to broaden antibiotics in EOS [3, 9].

Neonatal *E coli* meningitis comes with a risk of recurrence, therefore, a repeat LP to confirm sterilization is recommended. Therapy should be extended if sterilization is not yet achieved at 21 days [10, 11]. In the case presented here, sterilization was not assessed at the initial diagnosis of *E coli* meningitis nor at the end of therapy when the patient had stabilized. There are times when the decision is made to forgo proof of sterilization but this should only be considered in patients with uncomplicated infection and rapid clinical response to therapy; however, even that scenario is not a guarantee of bacterial clearance when it comes to neonatal *E coli* meningitis [10]. Unfortunately, our patient had intractable seizures and severe IVH concerning for ongoing infection. A repeat LP may have demonstrated evidence of continued infection resulting in prolongation of antibiotic therapy. As noted by *Vissing et al.*, neonatal *E coli* meningitis may be one of the clinical scenarios where pushing for shorter antibiotic courses and limiting invasive procedures is not providing benefit but increasing the chance for significant morbidity and mortality [10].

The recurrence of this patient’s *E coli* CNS infection resulted in global parenchymal infection with the formation of non-communicating cystic structures. Serially CSF sampling demonstrated notable pleocytosis and purulent fluid but bacterial gram stain and culture failed to detect organisms. PCR and NGS also failed to identify bacterial pathogens despite visual evidence on acridine orange stain of numerous, intact, rod-shaped bacteria. Due to extensive systemic and IV antibiotics, it is not surprising that the cultures were no growth. However, it was unexpected that serial CSF Gram stains, NGS, and PCR did not detect bacteria. No test is 100% sensitive and specific and all are subject to specific limitations. AO actually has a higher sensitivity compared to Gram stain but is not able to differentiate between gram-positive and -negative organisms, and is not used in the regular clinical microbiology workflow for CSF samples [12, 13]. However, unknown to many physicians, the newer NGS and PCR diagnostic techniques are not reliable in detecting pathogens when using samples with high numbers of inhibitory materials and cells present, i.e. pleocytosis [14, 15].

The addition of ciprofloxacin resulted in resolution of the CNS pleocytosis and bacterial rods on AO stain. Adjunct ciprofloxacin for neonatal *E coli* meningitis has been proposed for many years by some experts in France. However, there is not clear data demonstrating ciprofloxacin superiority to aminoglycosides or cephalosporins [16]. But, in this particular patient it may have provided improved bactericidal killing secondary to higher sensitivity or better CNS penetration compared to our initial regimen [17]. As the bacteria detected on AO did not grow in culture, we could not assess for the development of resistance after prolonged treatment with cefepime and aminoglycosides.

The infant presented here suffered severe consequences from recurrent, neonatal *E coli* CNS infection resulting in destruction of most of his brain parenchyma. Uncontrollable risk factors contributing to his poor outcome include his prematurity and infection with an *E coli* K1 strain. Premature infants even without CNS infection have a greater risk of long-term consequences [18]. K1 is a virulence factor that increases the ability of the bacteria to cross the blood brain barrier. However, initial treatment with cefepime rather than ampicillin and a repeat LP prior to stopping antibiotics may have altered the ultimate outcome of his infection. This case also illustrates the importance of understanding the limitations of classic sterilization testing as well as newer technologies. Identification of bacteria on AO stain stopped us from discontinuation of antibiotics prior to attaining true sterilization. The addition of ciprofloxacin allowed us to achieve sterilization in this patient though it is not entirely clear as to why it was successful. Key lessons from this patient include: (1) the use of AO stain when there are signs of continued infection despite negative GS and culture (2), always plan to repeat LPs to document sterilization in neonatal *E coli* meningitis unless the patient is clinically unstable, and (3) if patients are not clearing their CSF or have continued signs of clinical infection despite cefepime +/- an aminoglycoside, adjunct therapy with ciprofloxacin should be considered.

## Data Availability

The data presented in this report are available upon request from the corresponding author.

## Abbreviations

E coli: Escherichia coli
CNS: Central nervous system
PCR: Polymerase chain reaction
PICU: Pediatric intensive care unit
EOS: Early onset sepsis
LOS: Late onset sepsis
CSF: Cerebral spinal fluid
IVH: Intraventricular hemorrhage
LP: Lumbar puncture
IT: Intraventricular
MRI: Magnetic resonance imaging
NGS: Next-Generation sequencing
AO: Acridine orange
GBS: Group B Streptococcus
